# Intraoperative estimation of natural femoral anteversion from proximal femoral osseous orientation during total hip arthroplasty

**DOI:** 10.1186/s13018-024-05084-1

**Published:** 2024-09-28

**Authors:** Woo-Suk Lee, Soon-Phil Yoon, Ju Hyung Lee, Kwan Kyu Park, Kyoung Tak Kang, Byung-Woo Cho

**Affiliations:** 1 Department of Orthopedic Surgery, Gangnam Severance Hospital, Yonsei University College of Medicine, 211Eonju-ro, Gangnam-gu, Seoul, 06273 Korea; 2 Department of Orthopedic Surgery, Severance Hospital, Yonsei University College of Medicine, Seoul, Korea; 3 Department of Mechanical Engineering, Yonsei University, Seoul, Korea

**Keywords:** Total hip arthroplasty, Natural femoral anteversion, Intraoperative osseous orientation

## Abstract

**Background:**

The purpose of this study was to elucidate the relationship between the orientation of the osseous structure of the proximal femur encountered during total hip arthroplasty (THA) and preoperative femoral anteversion (FA).

**Methods:**

Three-dimensional models were constructed using full-length lower extremity computed tomography images from a total of 80 participants. Femoral neck cutting was performed at heights of 5, 10, and 15 mm relative to the lesser trochanter. Following neck cutting, the angles formed by the anterior outer cortex and posterior outer cortex with the posterior condylar line (PCL) were defined as the anterior cortical angle (ACA) and posterior cortical angle (PCA), respectively. Univariate linear regression analysis was conducted using the remaining measurements with FA as the dependent variable.

**Results:**

The mean age of the participants was 60.98 ± 10.82 years (males, 60.50 ± 11.36 years; females, 61.45 ± 10.37 years) (*p* = 0.697). All cortical angles and FA were larger in women compared to those in men. When comparing measurements by age groups, no statistically significant differences were observed. Univariate linear regression analysis with FA as the dependent variable showed statistical significance for all cortical angles. The adjusted R^2^ values were 0.711 (ACA5), 0.677 (ACA10), 0.572 (ACA15), 0.493 (PCA5), 0.574 (PCA10), and 0.446 (PCA15).

**Conclusion:**

Natural FA can be inferred from the anterior cortical angle (ACA) from femoral neck cutting plane observed during the THA procedure without preoperative images.

**Trial registration:**

Retrospectively registered.

## Introduction

In total hip arthroplasty (THA), the appropriate positioning of implants is crucial for preventing impingement and dislocation [1–3]. Traditionally, while the positioning of the acetabular cup and establishment of a safe zone have been emphasized [4], the introduction of the combined anteversion concept has underscored the significance of femoral stem anteversion as much as cup anteversion [5, 6]. Femoral stem anteversion, as a component of combined anteversion, influences the dislocation rate after THA [6] and impacts clinical outcomes following bipolar hemiarthroplasty [7]. According to Park et al., femoral stem anteversion is associated with preoperative femoral anteversion (FA) [8]. Therefore, assessing FA in advance is essential for a successful surgical outcome.

FA refers to the degree of torsion in the femur and is defined by the angle between the femoral neck axis (FNA) and posterior condylar axis projection [9]. While direct observation and measurement of osseous structures during surgery would be the most accurate method, it is impossible due to the inability to fully expose the distal femur. Consequently, most surgeons primarily rely on preoperative computed tomography (CT) images to determine angles by identifying the FNA and posterior condylar axis from single cross-sections. However, measurements using two-dimensional (2D) slice images without three-dimensional (3D) reconstructed models poses a high risk of error due to distortion caused by projecting the femur’s 3D anatomical geometry onto 2D slices. Various methods have been proposed to address this issue [10–14], but they still result in measurement errors. Kaiser et al. reported that CT measurement techniques could yield differences of up to 10º in measurements even for the same patient [15], while Schmaranzer et al. suggested differences of up to 20º in cases of excessive anteversion [16]. Furthermore, changes in patient positioning during the CT scan are known to influence measurements [13]. To overcome these limitations and accurately assess anteversion, some institutions may consider using robots or navigation systems, but accessibility to such equipment varies among facilities. Additionally, because FA is determined based on the anatomy of the distal femur, the absence of the entire femur in the CT scan makes it impossible to measure FA accurately. For instance, in patients who undergo hip or pelvic CT due to hip fractures, a challenging decision needs to be made to either undergo additional radiation exposure during a repeat CT scan to include the whole femur for determination of FA or blindly proceed with surgery without information on FA.

If we could infer FA from anatomical structures encountered during surgery, it could greatly aid in overcoming the challenges mentioned earlier in surgical planning. To our knowledge, no studies have reported on such methods. Therefore, we aimed to elucidate the relationship between the orientation of proximal femoral osseous structures encountered during THA and natural FA.

## Materials and methods

This retrospective study was conducted after obtaining approval from the Institutional Review Board (IRB) of our institution.

### Participant recruitment

We sequentially selected 40 men and 40 women aged ≥ 40 years who underwent THA at our institution and had preoperative full-length lower extremity CT imaging in 2023. The modeling was performed based on the side opposite to the surgical hip, and individuals who met the following criteria were excluded: (1) lack of inclusion of the femoral diaphysis in the CT images, (2) hindrance of anatomical identification due to hip joint arthritis or osteophytes, and (3) previous surgeries, dysplasia, congenital deformities, traumatic deformities of the femur, or implants in the femur.

### 3D model reconstruction and surgical simulation

**Fig. 1:**
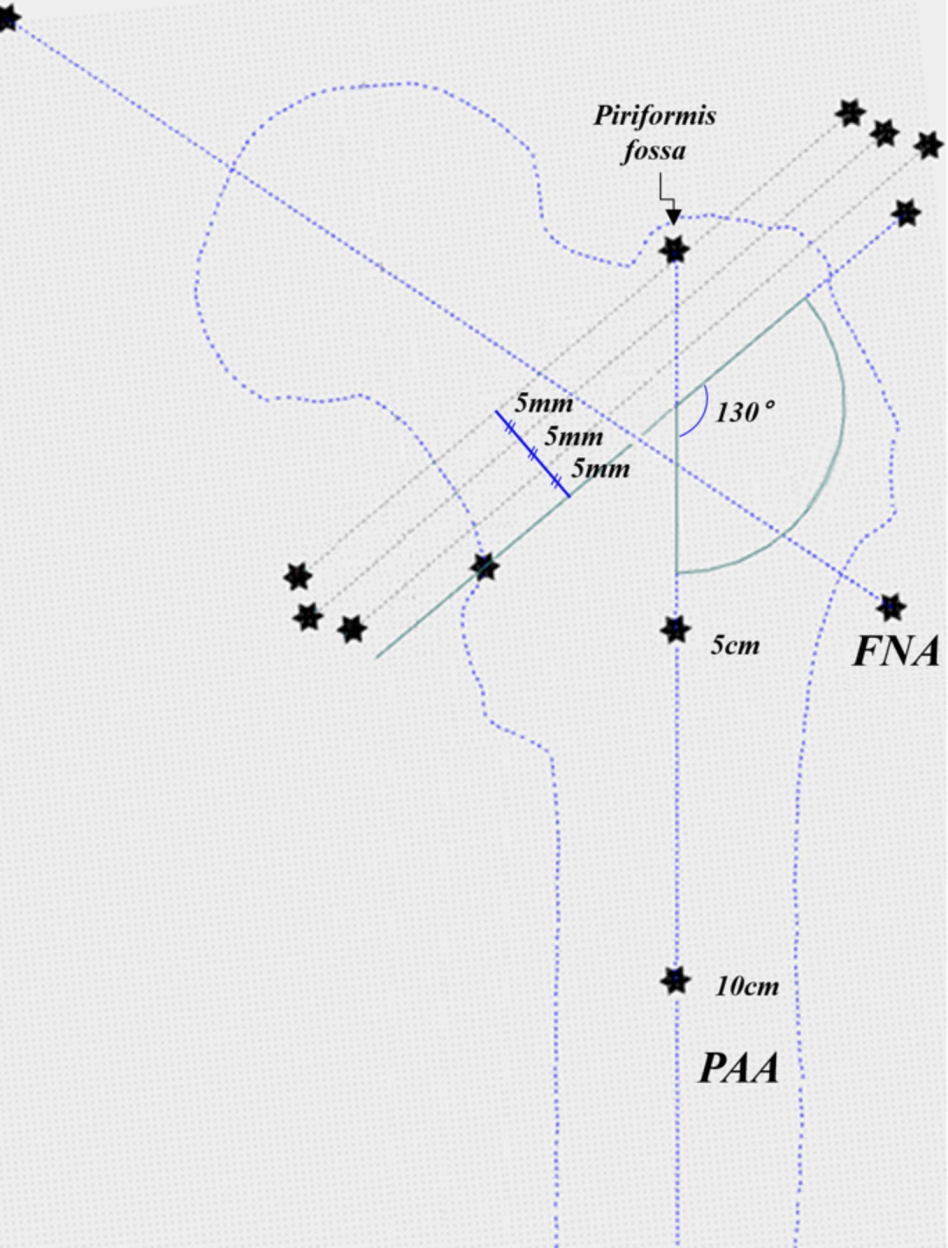
Overview of surgical simulation. FNA, femoral neck axis; PAA, proximal anatomical axis

3D model reconstruction and measurements were performed using Mimics and 3-Matic software (Materialize, Leuven, Belgium). The definitions of each landmark in the 3D model are as follows:

- FNA: A line connecting the center of the femoral head to the center of a circle tangent to the outer cortex of the femoral neck isthmus [17].
- Knee center: The point 1 cm above the intercondylar notch [18].
- Mechanical axis (MA) of femur: A line connecting the center of the femoral head to the knee center.
- Default position: The plane formed by the FNA and MA, parallel to the screen.
- Proximal anatomical axis (PAA): A line connecting the centers of the arcs tangent to the inner cortex of the femur at 5 cm and 10 cm distal to the piriformis fossa. This was defined for the purpose of simulating implant insertion.
- Femoral neck cutting: Cutting was performed similarly to the actual THA procedure (Depuy, Johnson & Johnson, Warsaw, IN, USA), along a plane with a 130º angle to the PAA and perpendicular to the screen from the default position. The method of determining the cutting position was similar to that of preoperative templating. Starting from the default position, three cuts were made at 5, 10, and 15-mm intervals above the lesser trochanter, based on the intersection of the medial cortical contour of the femoral neck and the superior aspect of the lesser trochanter (Fig. 1).

### Measurements

**Fig. 2:**
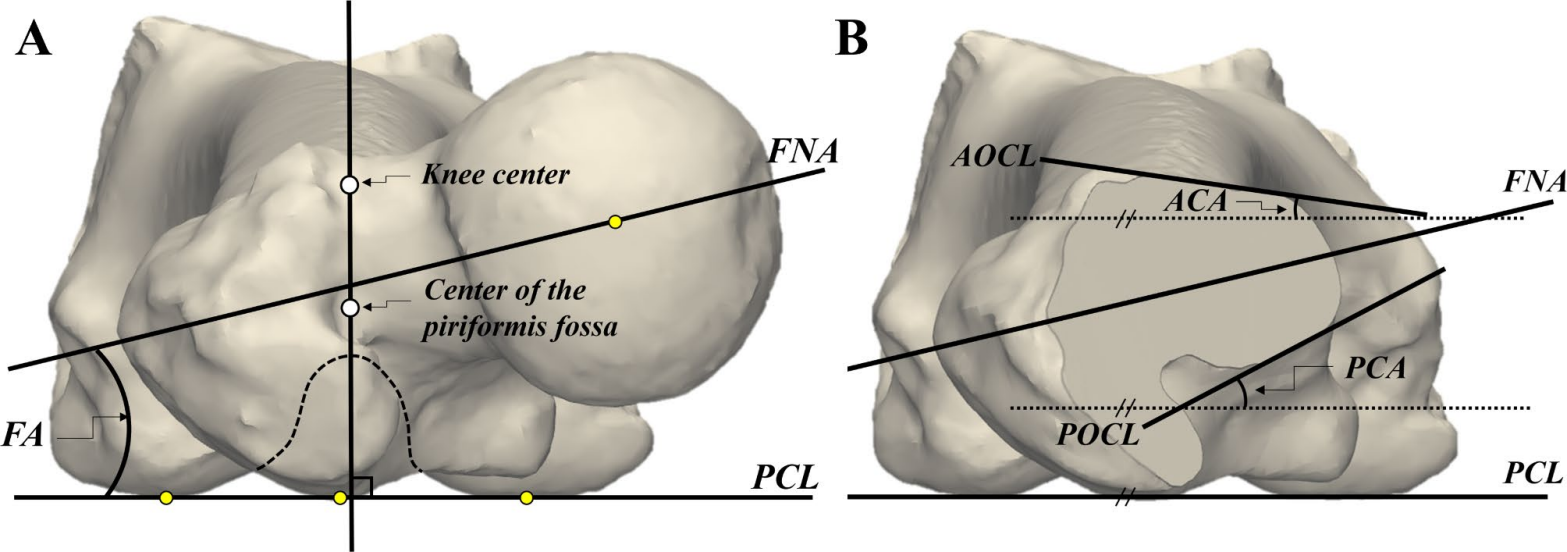
Definitions of position and measurements. FNA, femoral neck axis; PCL, posterior condylar line; FA, femoral anteversion; AOCL, anterior outer cortical line; POCL, posterior outer cortical line; ACA, anterior cortical angle PCA, posterior cortical angle

Measurement of FA was performed in a similar manner to the method described by Kingsley et al., with the participant in a table-top position [19]. Lines were drawn tangential to the posterior borders of the medial and lateral condyles of the distal femur, defining the posterior condylar line (PCL). The position was adjusted so that the most prominent point of the greater trochanter (GT) aligned with the PCL. At this position, a line perpendicular to the PCL was passed through the center of the piriformis fossa and the knee center. The definitions of each measurement at this position are as follows (Fig. 2):

- FA: The angle between the FNA and PCL.
- Anterior outer cortical line (AOCL): A line tangential to the anterior portion of the outer cortex at each cutting plane of the neck.
- Posterior outer cortical line (POCL): A line tangential to the posterior portion of the outer cortex at each cutting plane of the neck.
- Anterior cortical angle (ACA): The angle between the AOCL and PCL.
- Posterior cortical angle (PCA): The angle between the POCL and the PCL.

If the outer cortex is not an easily identifiable straight line, the line connecting the inflection points at both ends is defined as the outer cortical line. The ACA, PCA, and FA are defined with medial deviation from the PCL considered as positive (+). ACAs and PCAs were each measured at 5, 10, and 15-mm cutting intervals and named ACA 5, 10, 15 or PCA 5, 10, 15.

### Statistical analyses

An independent two-sample t-test was used to compare the measurements between sexes. Analysis of variance and Bonferroni’s post-hoc analysis were used to compare measurements across age groups. To analyze the correlation between cortical angles and FA, Pearson correlation analysis and univariate linear regression analysis were employed. The intra-observer and inter-observer reliabilities of the measurements were evaluated using intraclass correlation coefficients based on a total of 35 measurements, randomly extracted from 5 participants. Statistical analyses were performed using SPSS (version 25.0, IBM Inc., Armonk, NY, USA), and statistical significance was set at *p* < 0.05.

## Results

The mean age of the participants was 61.0 ± 10.8 years (men, 60.5 ± 11.4 years; women, 61.4 ± 10.4 years) (*p* = 0.697). All cortical angles and FA were significantly larger in women compared to those in men (Table 1).

**Table 1:** Differences in measurements between sexes

	Total (*n* = 80)	Men (*n* = 40)	Women (*n* = 40)	*p*-value
FA	12.6 ± 8.8	9.0 ± 7.3	16.2 ± 8.8	< 0.001
ACA5	-5.5 ± 11.1	-9.8 ± 9.0	-1.2 ± 11.5	< 0.001
PCA5	20.5 ± 11.4	17.0 ± 10.0	24.0 ± 11.8	0.006
ACA10	-7.1 ± 10.2	-10.8 ± 9.0	-3.5 ± 10.1	0.001
PCA10	17.7 ± 12.2	14.4 ± 10.1	21.0 ± 13.2	0.014
ACA15	-7.1 ± 10.6	-11.4 ± 8.7	-3.8 ± 11.0	0.001
PCA15	16.7 ± 12.4	13.1 ± 10.1	20.3 ± 13.6	0.009

FA, femoral anteversion; ACA, anterior cortical angle; PCA, posterior cortical angle

*The numbers following ACA or PCA indicate the cutting levels of the planes where measurements were taken

When the participants were divided into four age groups for comparison, significant differences were observed only in PCA 15 in participants aged 51 − 60 years, while no significant differences were observed among the other groups (Table 2).

**Table 2:** Differences in measurements across the age groups

	< 50 (*n* = 12)	51 − 60 (*n* = 28)	61 − 70 (*n* = 21)	> 70 (*n* = 19)	*p*-value
FA	7.4 ± 8.8	15.2 ± 9.2	11.6 ± 8.5	13.3 ± 7.5	0.720
ACA5	-10.1 ± 9.6	-3.0 ± 11.7	-7.1 ± 10.7	-4.6 ± 11.4	0.260
PCA5	15.1 ± 11.8	22.9 ± 10.4	18.6 ± 10.4	22.4 ± 13.0	0.170
ACA10	-11.7 ± 10.2	-6.0 ± 9.8	-7.2 ± 11.1	-5.8 ± 9.6	0.384
PCA10	14.3 ± 12.1	22.3 ± 11.7	13.6 ± 10.3	17.6 ± 13.4	0.058
ACA15	-11.8 ± 10.2	-6.9 ± 9.6	-7.4 ± 11.9	-6.3 ± 11.0	0.529
PCA15	15.2 ± 12.6	22.5 ± 12.2*	11.2 ± 10.5*	15.2 ± 11.9	0.012

FA, femoral anteversion; ACA, anterior cortical angle; PCA, posterior cortical angle

*Bonferroni post-hoc test results indicate a statistically significant difference between the 51 − 60 and 61 − 70 year age groups

*The numbers following ACA or PCA indicate the cutting levels of the planes where measurements were taken

**Fig. 3:**
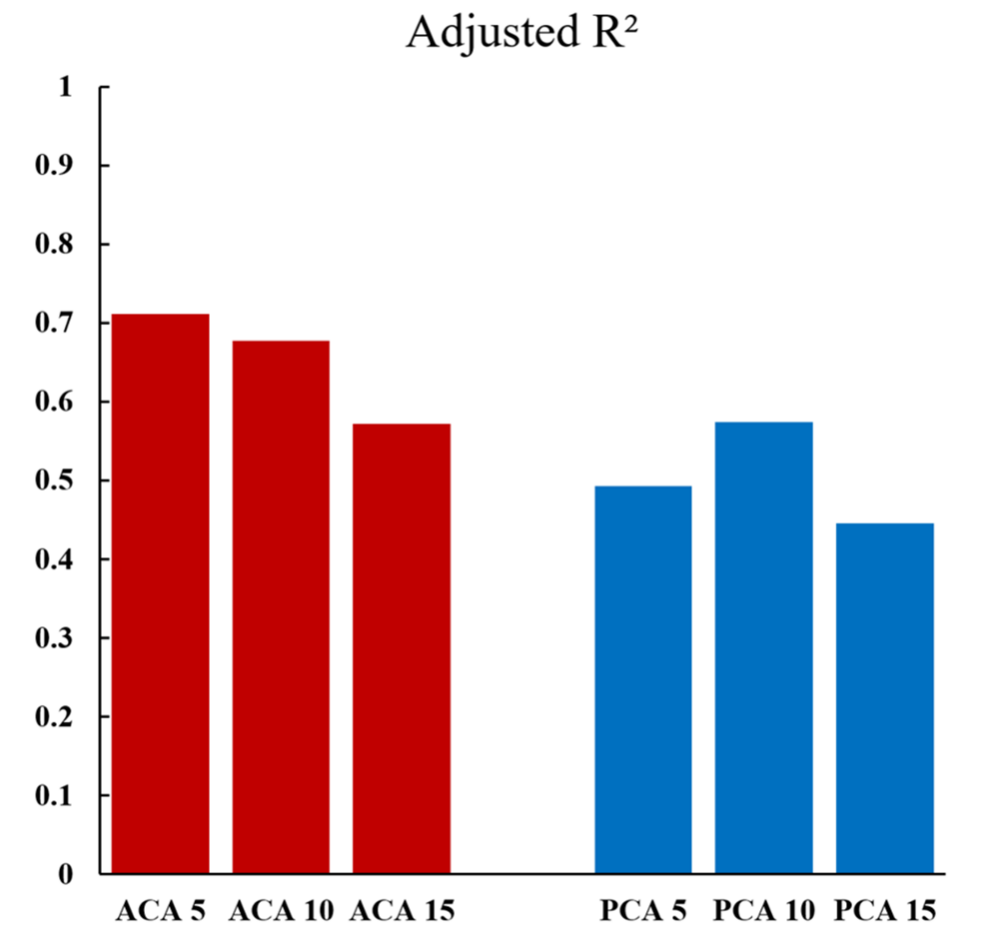
The adjusted R^2^ values from the univariate linear regression analysis conducted with femoral anteversion as the dependent variable. ACA, anterior cortical angle; PCL, posterior cortical angle

**Fig. 4:**
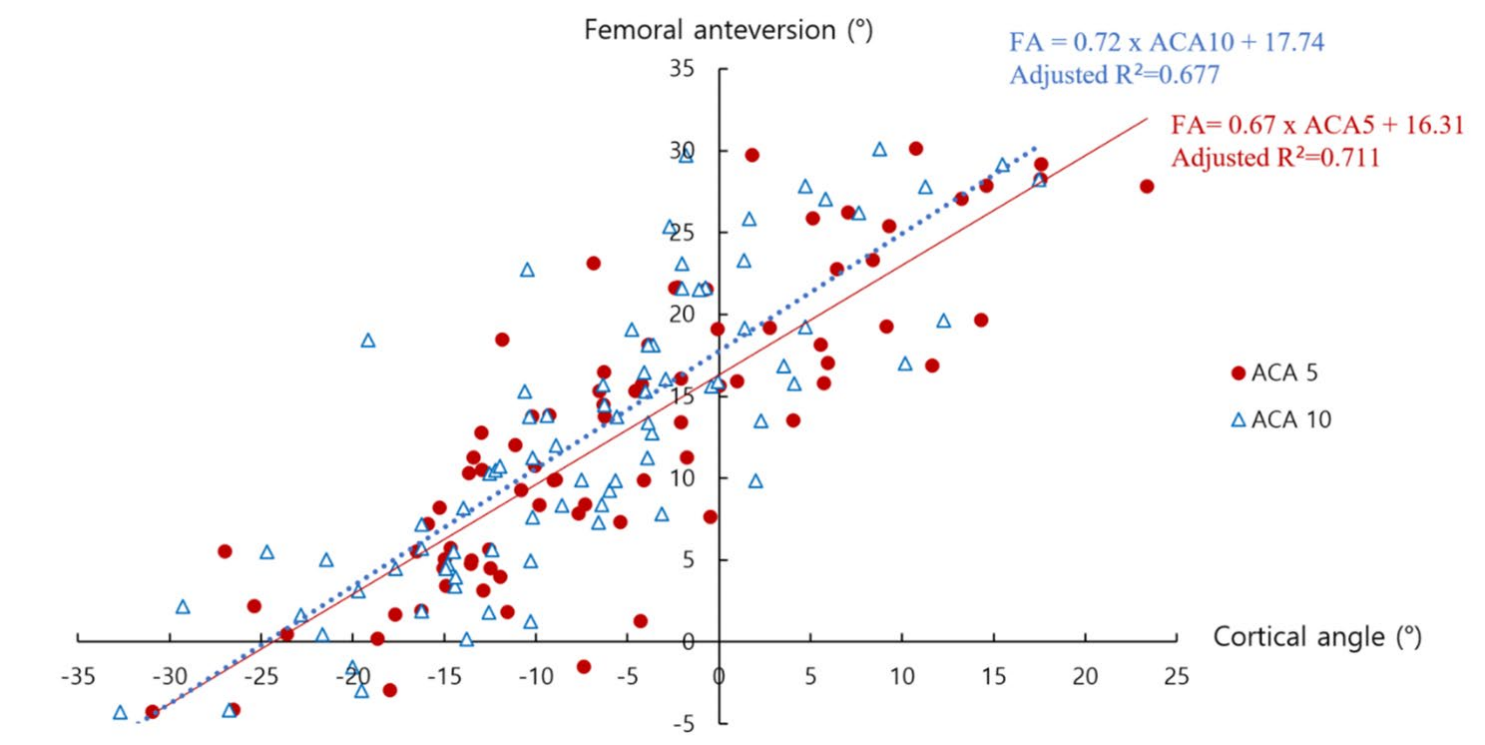
Relationship between femoral anteversion and intraoperatively measured anterior cortical angles. FA, femoral anteversion; ACA, anterior cortical angle

In univariate linear regression analysis with FA as the dependent variable, all cortical angles showed statistical significance. The adjusted R^2^ values were 0.711 (ACA5), 0.677 (ACA10), 0.572 (ACA15), 0.493 (PCA5), 0.574 (PCA10), and 0.446 (PCA15) (Fig. 3). A strong positive correlation was demonstrated between FA and both ACA5 and ACA10, with adjusted R^2^ values of 0.6 or higher. The regression equations were as follows: FA = 0.67 x ACA5 + 16.31 and FA = 0.72 x ACA10 + 17.74 (Fig. 4). The ranges of intra-observer and inter-observer reliabilities were 0.997-1.000 and 0.899-1.000, respectively.

## Discussion

The most important finding of this study was that FA can be inferred solely from the osseous structure of the proximal femur. In other words, during the traditional THA procedure, assessing the position of the anterior outer cortex on the cutting plane allows for an estimation of the degree of torsion of the entire femur. If the neck cutting is within 10 mm of the lesser trochanter, approximately 16 − 18º of FA can be assumed when the ACA is approximately 0º. When the ACA is approximately − 10º degrees, it may indicate approximately 10º of femoral anteversion.

Femoral torsion begins at approximately gestational age (GA) 8 weeks [20] and increases to approximately 30 − 40º during the fetal period [21–23]. According to the study by Zhao et al. in which serial sections of the hip and thigh were used from 34 embryos and fetuses, fetal period femoral torsion primarily occurred in the distal femoral shaft rather than at the trochanters [20]. Additionally, the lesser trochanter (LT) emerges on the opposite side of the GT where the iliopsoas inserts at approximately GA 9–10 weeks and gradually shifts to a position resembling that of adults by GA 12 weeks [20]. Therefore, femoral torsion during development does not distort proximal geometry, leading to the assumption that the outer cortices of the femoral neck have similar orientations to the femoral neck itself. Moreover, the anterior cortex of the femoral neck is further from the location where the LT develops and moves compared to that of the posterior outer cortex; therefore, it is thought to be less distorted. Our study results can be adequately explained by these embryological findings.

Traditionally, during THA, the implantation of the acetabular cup precedes that of the femoral stem.However, in THA procedures using cementless stems, a stem-first approach is necessary for the combined anteversion technique [24]. This approach is necessary because the range of FA varies [9], and since Park et al. reported that the anteversion of the stem follows the native FA [8], there is a risk of not achieving the target anteversion value if the cup is implanted first. However, the stem-first procedure poses surgical inconveniences for the following reasons: First, once the stem or rasp is inserted, it may obstruct the view during acetabular bone preparation or interfere with the entry of the acetabular reamer. Second, even with only femoral broaching, bone bleeding from the femoral canal can occur during acetabular bone preparation. To overcome these limitations, Masumoto et al. also reported the cup-first procedure using cementless stems [25]. Based on our research findings, FA can now be inferred solely after neck cutting, allowing for acetabular cup insertion as the initial step. In other words, using our method, cup-first THA with combined anteversion technique may be possible, even with cementless stems.

In this study, we compared FA and cortical angles by sex and age. When comparing the sexes, women exhibited larger values than men, consistent with findings in the existing literature [26, 27]. However, no significant differences were observed between age groups, which aligns with results of some studies reporting no age-related differences [28] while others suggest a decrease in anteversion with age [29, 30]. These discrepancies may stem from variations in the age range of registered patients and measurement methods [15, 16]. Differences between 3D models and 2D slice cuts can occur, and errors due to variations in measurement methods may also arise in studies utilizing 3D models [28, 31]. Despite efforts to minimize measurement errors by adopting definitions similar to those used in previous studies that utilized actual bone, further studies using a larger number of models are warranted to more comprehensively address this issue.

Applying our research findings to actual surgery encounters several limitations. First, intraoperative measurements may not be as precise as simulations. This limitation is not unique to our study but is common in most studies involving intraoperative measurements. Several studies comparing intraoperative anteversion measures with postoperative CT images have shown significant discrepancies, with varying differences across studies [32–34]. Second, our method relies on measuring all angles relative to PCL, which may not be accurately discernible during surgery. The posterolateral approach typically aligns the lower leg vertically to assume that the PCL and the ground are horizontal, which may be influenced by factors such as the patient’s knee status and laxity. Therefore, rather than applying the formula we proposed, evaluating the angles based on easily assessable angles, such as when the ACA is 0º or -10º, as mentioned in the first paragraph in the discussion section would be more practical and useful. Third, this study has a retrospective design and a small sample size, making it difficult to eliminate selection bias. A larger sample size would be necessary to better represent a broader population.

In conclusion, FA can be inferred from the osseous structure of the proximal femur observed during the THA procedure without preoperative images. As a result, significant assistance can be provided in surgical planning, such as enabling the cup-first procedure in the combined anteversion technique with a cementless stem.

## Data Availability

No datasets were generated or analysed during the current study.
